# Limb asymmetries persist 6 months after anterior cruciate ligament reconstruction according to the results of a jump test battery

**DOI:** 10.3389/fmed.2024.1303172

**Published:** 2024-02-20

**Authors:** Claudio Legnani, Matteo Del Re, Giuseppe M. Peretti, Enrico Borgo, Vittorio Macchi, Alberto Ventura

**Affiliations:** ^1^IRCCS Istituto Ortopedico Galeazzi, Milan, Italy; ^2^Department of Biomedical Sciences for Health, University of Milan, Milan, Italy

**Keywords:** anterior cruciate ligament, ACL reconstruction, return to sport, vertical jump, test battery

## Abstract

**Objectives:**

Test batteries used to assess a patient’s return-to-sports (RTS) following anterior cruciate ligament reconstruction (ACLR) are currently undergoing continual development, although no consensus exist on tests to be administered to athletes before allowing return to play. A simple standardized jump test battery was developed to objectively evaluate knee function following ACLR, thereby aiding in RTS decision-making.

**Methods:**

Thirty-three patients who underwent ACLR were prospectively assessed pre-operatively, 6, and 12 months after surgery. Knee function was assessed using a device for optical detection using a test battery consisting of three jump tests: monopodalic countermovement jump (CMJ), drop jump, and monopodalic side-hop. Limb symmetry index (LSI) was reported for all tests at all time points. LSI ≥90% was defined as RTS criteria.

**Results:**

At 12-month evaluation, mean LSI significantly improved compared to 6-month follow up (*p* < 0.01), and also compared to baseline (*p* < 0.01), reporting a mean value of 92.6% for CMJ, 90.6 for drop jump and 96.9% for side hop test. Most patients fulfilled the RTS criteria 12 months after surgery (LSI ≥90%). The percentages of patients demonstrating LSI ≥90% at 6 months was 7/33 (21.2%) for CMJ, 12/33 (36.4%) for drop jump, and 11/33 (33.3%) for side-hop test. One year after surgery, percentages grew up to 66.6% (22/33), 63.6% (21/33), and 81.8% (27/33) respectively.

**Conclusion:**

Six months after ACLR, knee functional performance was unsatisfactory in most patients, whereas a significantly higher percentage of patients met RTS criteria 1 year after surgery. The results of the jump test battery proposed in this study support the idea that timing for resumption of cutting and pivoting sports should be delayed later than 6 months, as still limb asymmetries persist at this time point.

## Introduction

Ruptures to the anterior cruciate ligament (ACL) are among most frequently encountered injuries in subjects practicing cutting and pivoting sports and in active patients experiencing knee instability is usually addressed surgically (1, 2).

The ability of patients who have had ACL reconstruction to resume sport and recreational activities is a major concern, and the orthopaedic community is working to establish safe criteria for this, as well as to reduce complications like graft re-injuries, which are higher in the young population (3–5).

Creating criteria to direct return to sport (RTS) decision-making is crucial. Therefore, there is general agreement that a thorough test battery, including an objective physical evaluation, is required to clear sportsmen to RTS (6). Studies demonstrated that deficiencies in lower-limb neuromuscular control and knee strength are the two main characteristics that can affect a sportsman’s capacity to RTS, and can be assessed with the help of complete test batteries (6, 7).

Test batteries used to assess patient RTS following ACL reconstruction are currently undergoing ongoing development and getting closer to provide reliable and reproducible information (8).

Functional assessments have been devised to track patients’ ability to resume athletic activities after ACL restoration (9). By assessing explosive strength, power, and responsiveness, vertical jump tests have shown to be able to accurately identify functional asymmetries between limbs and evaluate knee biomechanics (10–13). Knee function recovery following ACL reconstruction is often assessed using the limb symmetry index (LSI), which expresses muscular strength, jump performance, and altered knee kinematics as percentages of contralateral limb values (14). Test batteries used to assess a patient’s RTS following ACL reconstruction are currently undergoing continual development, although no consensus exist on tests to be administered to athletes before allowing return to play, and at present used RTS criteria after ACL surgery are still arbitrary and often time-based.

In the current study, we aimed to identify knee functional deficits after ACL reconstruction with the help of a jump test battery, to allow to determine patients’ capability to resume sporting activity. The hypothesis was that ACL reconstruction improves LSI measured during jump tests.

## Patients and methods

### Patients recruitment

The present study included 33 non-professional athletes who had ACL injury and were subjected to ACL surgery between January and December 2021. Participants were prospectively evaluated up to 12 months after ACL surgery. Primary unilateral ACL reconstruction; age between 18 and 50 years at surgery; recreational or competitive engagement in sports; and the same postoperative rehabilitation regimen were inclusion criteria. The following were listed as exclusion criteria: a previous ligament surgery on the ipsilateral or opposite limb; concurrent surgical operations other than those necessary to treat meniscal disease; pregnancy; and an inability to pass clinical and functional testing.

Overall, thirty-one male and two female were included in the present study. Age at operation was 34.0 years on average (SD: 11.5). Mean body mass index (BMI) was 25.4 (SD 3.2). Mean time between an injury and surgery was 2.9 months (SD: 1.2) (Table 1).

**Table 1:** Patient demographics and anthropometric data.

No. of patients	33
Gender	
Male	31
Female	2
Mean age at surgery (SD) (yr)	34.0 (11.5)
Mean time from injury to surgery (SD) (mo)	2.9 (1.2)
Mean BMI (SD)	25.4 (3.2)

SD, standard deviation; BMI, body mass index.

Prior to beginning this investigation, institutional review board approval was received from IRCCS San Raffaele Hospital in Milan, Italy, (IRB number: 57/INT/2020), and each subject provided their informed consent.

### Surgical technique and rehabilitation protocol

Arthroscopic assisted ACL reconstruction using doubled autologous hamstring graft was performed in all patients as previously described (3). All patients underwent brace-free rehabilitation beginning the day after surgery, which included isometric exercises, early recovery of extension, and walking with crutches for the first 3 weeks. Swimming and indoor cycling were permitted after 12 weeks, while a jump technique training and plyometric exercise routine was introduced after 5 months. During outpatient and inpatient rehabilitation, surgeons and physiotherapists closely observed every patient to track their development and compliance with the protocol.

### Follow-up assessment

An infrared optical acquisition device (OptoGait; Microgate, Bolzano, Italy) was used to assess vertical jump tests performance. Previous studies demonstrated validity and reliability of this device in measuring spatial–temporal parameters (15, 16). Patients had been told to warm up for 10 min while conducting practice trials. The test battery was composed by a monopodalic counter movement jump (CMJ) test, a drop jump and a monopodalic side hop test. The intact limb was tested first, then the injured. Except for the side hop test, which was carried out once for each leg, each functional test was completed three times. Each jumping performance was separated by a sufficient amount of recovery time. Test results were determined as the average of the trials run, and they were recorded as flight time (in milliseconds) and distance (in centimeters). During monopodalic jumps, the LSI was reported as a percentage of test performance on the unaffected leg compared to the healthy limb.

### Statistical analysis

Data were analyzed using Graphpad Prism v8.0 (Prism Software, La Jolla, CA, United States). According to the results of Shapiro–Wilk test, when non-normal data distribution was present, differences between follow-ups were assessed using Friedman’s test and Dunn’s *post hoc* test for pairwise comparisons; one-way ANOVA with Tukey’s *post hoc* test was used for normally distributed data. *p* values <0.05 were considered statistically significant.

## Results

**Figure 1:**
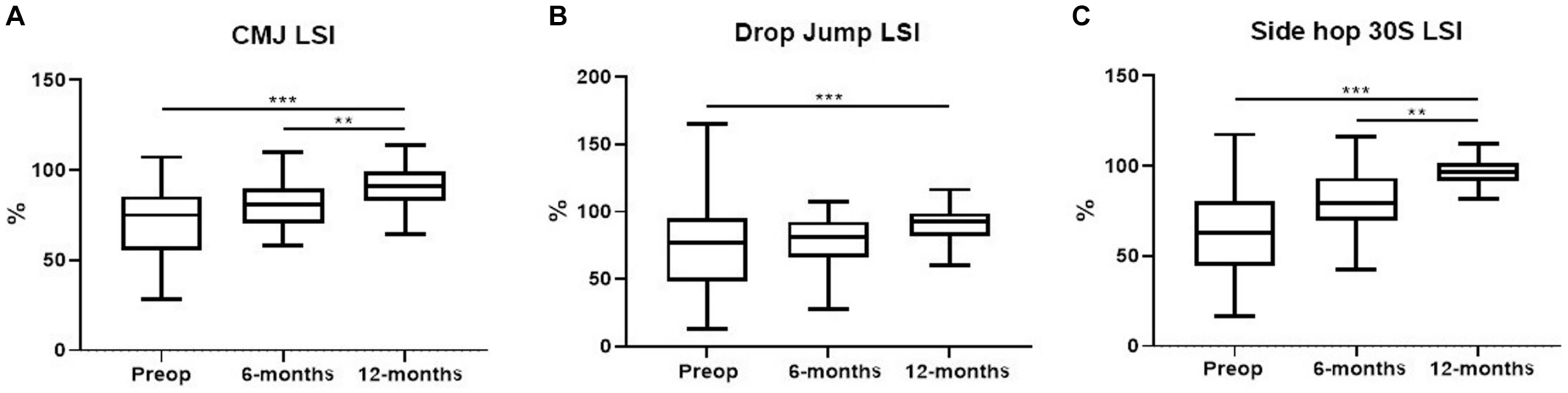
Box-plots showing LSI of monopodalic CMJ **(A)**, drop jump **(B)**, and side hop test **(C)** performances pre-operatively, 6 and 12 months follow-up after surgery. The lowest bar represents the minimum value, the bottom and top of the boxes represent the interquartile range (25th and 75th percentiles), and the top bar represents the maximum value. **p < 0.01, ***p < 0.001.

At the 12-month evaluation (92.6% vs. 72.3%, respectively; *p* < 0.001) and 6-month follow-up (81.7%, *p* < 0.01), CMJ LSI shown improvements over baseline. Between the baseline and the 6-month evaluation, no differences were reported (Figure 1A).

Drop jump LSI improved from the baseline at the 12-month evaluation (*p* < 0.001); during the follow-up, the mean value improved from 76.6% (SD: 32.4) to 90.2% (SD: 14.7; *p* < 0.001). There were no differences between the 6- and 12-month evaluations (*p* = n.s.) or between the 6-month evaluation and the baseline (Figure 1B).

Side Hop 30S LSI demonstrated improvements at 6-month follow-up (80.5%, *p* < 0.01) and 12-month evaluation compared to baseline (96.9% vs. 62.4%, *p* < 0.001). Between the baseline and the 6-month assessment, no differences were found (*p* = n.s., Figure 1C).

The percentages of patients demonstrating LSI ≥90% at 6 months was 7/33 (21.2%) for CMJ, 12/33 (36.4%) for drop jump, and 11/33 (33.3%) for side hop test. One year after surgery, percentages grew up to 66.6% (22/33), 63.6% (21/33), and 81.8% (27/33) respectively.

## Discussion

In addition to widely used and validated PROMs, other functional test batteries have been developed to evaluate RTS following ACL reconstruction (17–19). According to earlier studies, a vertical jump can accurately assess the strength, explosive power, and neuromuscular control of the quadriceps (10–13, 20, 21). The level of functional recovery following ACL surgery was assessed in the current study using a series of vertical jump tests to find asymmetries between limbs both before and after surgery.

An improvement in the LSI measured during the CMJ, drop jump, and side hop tests was seen a year following surgery. Most patients had inadequate knee functional performance 6 months after ACL reconstruction, however considerably more patients met RTS criteria 1 year following surgery.

Our results showed that the average LSI recorded during the jumping performances ranged from 77.3 to 81.7% at 6 months post-surgery, and overcame 90% a year later following ACLR. LSI > 90% is typically recommended as the cutoff score to enable patients to RTS (22), although its validity is debated since there is a chance that patients would overestimate their performance because of concurrent deterioration of function in the uninjured limb (23). For these reasons, several articles have suggested that recovery time for returning to sports should be at least 9 months following surgery (24–26). Nine months after ACLR, a previous study by Read et al. reported between-limb asymmetries in jumping parameters in professional soccer players, confirming the possibility of a compensating method to unload the affected limb during the vertical jump test (27).

The results of the test battery proposed in our study support the idea that timing for resumption of cutting and pivoting sports should be delayed later than 6 months, as still limb asymmetries persist at this time point.

Our findings show that there were no differences between baseline and the 6-month evaluation for any jump tests. Accordingly, muscle coordination recovery does not start happening until 6 months following surgery, and 1 year later, explosive leg power and neuromuscular control tend to reach their pre-surgical states rather than dramatically improving. Failure to meet the six-month return-to-sports criterion may preclude a successful RTS performance and may raise the risk of reinjury. Our results support the notion that the window for the return to cutting and pivoting sports should be extended past 6 months since still present limb asymmetries still exist at this time (24–26).

The relatively small sample size of the current study is one of its limitations since it makes it harder to identify subtle changes between groups when it comes to specific metrics. OptoGait was chosen because it is a straightforward, inexpensive tool that is simple to use in a clinical context and enables accurate evaluations of functional ability. We understand that a variety of factors affects a person’s ability to jump, and another study limitation is the use of jump height as a measure of neuromuscular recovery after ACL surgery. To overcome these issues, a standardized protocol encompassing CMJs, drop jumps and side-hop tests has been developed to allow to better investigating jump performance. In addition, since many sporting activities include unilateral propulsion in both vertical and horizontal directions, unilateral evaluation seems to more accurately capture the power related to these specific gestures. As a further limitation, we acknowledge that female knee kinematics can differ from males due to anatomy, kinematics, and hormonal status, thus potentially affecting the outcomes. Our findings cannot be applied to females because the patients’ recruitment ratio of male to female was biased in favour of the male sex. The current study aims to fill the current gap in the literature about objective standards for judging athletes’ readiness to RTS following ACL reconstruction. The association between the factors involved in returning to sports activities must be investigated in future studies with larger cohorts and other tests, allowing decision-making for a safer RTS after ACL surgery.

## Conclusion

Six months after ACL reconstruction, limb asymmetries were detected in most patients according to the jump test battery proposed in this study, whereas the average LSI recorded during the jumping performances reached 90% 12 months after ACLR. These results validate the notion that timing for RTS should be delayed later than 6 months after surgery.

## Data availability statement

The raw data supporting the conclusions of this article will be made available by the authors, without undue reservation.

## Ethics statement

The studies involving humans were approved by IRCCS San Raffaele Hospital, Milan Italy. The studies were conducted in accordance with the local legislation and institutional requirements. The participants provided their written informed consent to participate in this study.

## Author contributions

CL: Conceptualization, Data curation, Formal analysis, Investigation, Validation, Writing – original draft, Writing – review & editing. MD: Data curation, Formal analysis, Investigation, Software, Writing – review & editing. GP: Funding acquisition, Project administration, Resources, Supervision, Visualization, Writing – review & editing. EB: Data curation, Formal analysis, Methodology, Software, Writing – review & editing. VM: Data curation, Formal analysis, Methodology, Software, Writing – review & editing. AV: Funding acquisition, Project administration, Resources, Supervision, Visualization, Writing – review & editing.
